# Integrating Morphological, Sensory, and Chloroplast Genetic Diversity Reveals Regional Structuring and Domestication Potential of *Mimusops zeyheri* Sond. in South Africa

**DOI:** 10.3390/biology15161364

**Published:** 2026-08-11

**Authors:** Christeldah Mkhonto, Peter Tshepiso Ndhlovu, Luambo Jeffrey Ramarumo, Wilfred Otang Mbeng, Luxon Nhamo, Sylvester Mpandeli, Salmina Ngoakoana Mokgehle

**Affiliations:** 1 School of Biology and Environmental Sciences, Faculty of Agriculture and Natural Sciences, University of Mpumalanga, Private Bag X11283, Mbombela 1200, South Africa; 201707659@ump.ac.za (C.M.); tshepiso.ndhlovu@ump.ac.za (P.T.N.); luambo.ramarumo@ump.ac.za (L.J.R.); wilfred.mbeng@ump.ac.za (W.O.M.); 2 Water Research Commission (WRC), Private Bag X03, Gezina, Pretoria 0031, South Africa; luxonn@wrc.org.za (L.N.); sylvesterm@wrc.org.za (S.M.); 3 School of Agricultural Sciences, Faculty of Agriculture and Natural Sciences, University of Mpumalanga, Private Bag X11283, Mbombela 1200, South Africa

**Keywords:** *Mimusops zeyheri*, genetic diversity, sensory evaluation, chloroplast markers, ecological variation, domestication, indigenous fruit trees

## Abstract

*Mimusops zeyheri* Sond. is an underutilized indigenous fruit tree with significant nutritional, ecological, and socio-economic value in southern Africa. However, its domestication potential remains constrained by a limited understanding of intraspecific variation across geographically distinct populations. This study integrated morphological, sensory, and chloroplast genetic analyses to assess the diversity and population structuring of *M. zeyheri* in two regions of South Africa (Limpopo and Mpumalanga). A total of 40 trees (20 per region) were evaluated for fruit, nut, and leaf traits, while sensory attributes were assessed by 100 participants using a 9-point hedonic scale. Genetic diversity was examined using the chloroplast markers *matK* and *trnH–psbA*. Significant regional differences were observed in reproductive morphology, with Mpumalanga populations producing larger fruits (29.41 ± 0.61 mm) and nuts (2.11 ± 0.36 cm) than those from Limpopo. In contrast, sensory evaluation revealed consistently higher preference scores for Limpopo fruits across all attributes, particularly taste, aroma, and overall acceptability (mean scores 7.7–8.0 vs. 5.0–5.4). Genetic analyses identified two major chloroplast lineages corresponding broadly to geographic origin, alongside rare haplotypes indicating localized divergence. Leaf morphology remained largely conserved across regions. The combined evidence demonstrates strong regional structuring driven by both environmental gradients and historical genetic divergence. Importantly, the decoupling of fruit size and sensory preference highlights the need to prioritize quality traits in domestication strategies. These findings point to a promising domestication direction, through crossbreeding superior-tasting Limpopo genotypes with larger-fruited Mpumalanga genotypes to combine consumer-preferred sensory quality with an improved fruit size. These findings provide a foundation for conservation planning and the selection of superior genotypes for the development of *M. zeyheri* as a high-value indigenous fruit crop.

## Simple Summary

Domesticating underutilized indigenous fruit trees such as *Mimusops zeyheri* requires a clear understanding of how populations vary across their natural range. This study compared trees from two regions of South Africa, Limpopo and Mpumalanga, examining fruit, nut, and leaf measurements, consumer sensory ratings, and chloroplast DNA markers that trace maternal ancestry. Mpumalanga trees produced larger fruits and nuts while Limpopo fruits were rated consistently higher for taste, aroma, and overall acceptability, and the two regions carried distinct genetic lineages. These findings indicate that fruit size and taste are influenced by separate underlying factors. This knowledge can guide the selection of parent trees for breeding programmes aimed at developing *M. zeyheri* into a resilient, high-quality indigenous crop.

## 1. Introduction

Variation within a species forms the basis of its capacity to adapt, persist, and evolve under changing environments. In fruit-bearing trees, the interaction between genetic make-up, morphological form, and sensory attributes determines both ecological fitness and human value [1]. For many indigenous African fruit species, this intricate web of variation remains largely unexplored. *Mimusops zeyheri* Sond. is a fitting example. Valued by rural communities for its sweet, aromatic fruits and durable timber, it continues to be cultivated informally despite its considerable ecological and nutritional potential [2].

Underutilized indigenous fruit trees such as *M. zeyheri* are increasingly recognized as critical resources for food security, nutritional diversity, and climate resilience in semi-arid and rural landscapes, and could improve rural livelihoods if properly packaged, branded, and promoted. Yet how the species varies genetically and phenotypically across regions, and how this variation relates to taste and consumer preference, remains poorly understood. In semi-arid regions where conventional staple crops are increasingly failing under erratic rainfall and rising temperatures, unlocking the domestication potential of resilient indigenous species such as *M. zeyheri* is critical for strengthening agricultural resilience and diversifying rural food systems.

In recent years, researchers have become increasingly interested in underutilized fruit trees like *M. zeyheri*, as they seek hardy, nutrient-rich fruits suitable for semi-arid and changing climates [2,3]. The fact that the majority of these underutilized indigenous fruit trees are found in rural areas provides an opportunity for smallholder farmers to focus on these crops if there are market opportunities. These underutilized indigenous crops adapt easily under different environments, and they are easy to manage. Some of the community members use these underutilized crops trees for medicinal purposes and rituals on an annual basis. These species are genetic reservoirs that can help guide future breeding and conservation measures. However, the promise and potential of *M. zeyheri* cannot be realized without a thorough analysis of its internal diversity. Multiple traditional research studies have documented mainly ethnobotanical uses and nutritional attributes of the species in isolation [2,4,5]. For example, Omotayo et al. [2] focused primarily on the nutritional composition and resource value of *M. zeyheri* fruit, while Lubisi et al. [4] examined patterns of local utilization and population perceptions in the Vhembe Biosphere Reserve; neither study linked genetic variation with phenotypic or sensory traits. This gap, the absence of an integrated genetic–morphological–sensory perspective, is the specific problem this study addresses. Assessing genetic diversity within *M. zeyheri* populations provides an essential foundation for their conservation and improvement. Although the species is increasingly recognized for its nutritional and ecological value, molecular-level studies remain scarce. Limited studies have applied DNA-based markers to explore variation among its genotypes, and even fewer have linked that variation to fruit quality or adaptation. Without this knowledge, breeding and domestication efforts remain largely empirical. In any plant improvement programme, progress depends on the extent of genetic variation and how it can be utilized. The wider the genetic base, the greater the chance of combining traits that enhance yield, resilience, or fruit quality [6]. Importantly, understanding genetic diversity is not only essential for conservation but also forms the basis for identifying superior genotypes that can be selected and propagated in domestication and crop improvement programmes.

Within *M. zeyheri*, this diversity underpins more than adaptation; it is the key to nutritional and phytochemical potential. Variability in genes controlling the synthesis of sugars, minerals, and secondary metabolites likely explains the differences in fruit sweetness, colour, or antioxidant activity seen across populations [7,8]. Understanding these genetic foundations allows researchers to identify genotypes that are both agronomically promising and nutritionally superior. Populations with distinctive genetic traits may also exhibit biochemical advantages, including potentially higher concentrations of phenolics, vitamins, or essential minerals, which contribute to the species’ dietary and medicinal value [9,10]. Given the contrasting ecological conditions between regions such as Limpopo and Mpumalanga, ecological variation is likely to play a significant role in shaping both phenotypic traits and underlying genetic structure within the species.

To investigate these relationships, this study combined molecular, morphological, and sensory analyses. *Mimusops zeyheri* leaf, fruit, and nut characteristics were recorded to document visible variation between and within regions. DNA was extracted from leaf tissue to evaluate genetic diversity and population structure, providing a molecular perspective on differentiation that morphology alone cannot explain. At the same time, sensory evaluation captured how people experience the fruits, their sweetness, aroma, and texture, offering a human dimension that links genotype to perception. Such an integrated approach helps clarify how *M. zeyheri* has adapted to distinct environments and points toward its potential for selection, propagation, and eventual domestication as a high-value indigenous fruit tree. In addition to agronomic traits, sensory attributes such as taste, aroma, and texture are critical determinants of consumer acceptance and market potential and therefore play a central role in the successful domestication of indigenous fruit species. Taken together, these gaps highlight the need for a more integrative approach that captures multiple dimensions of variation within the species.

To address these gaps, this study adopts an integrated approach combining morphological, sensory, and molecular analyses to evaluate variation within *Mimusops zeyheri* populations from two contrasting agro-ecological regions of South Africa. Specifically, fruit, nut, and leaf traits were quantified to capture phenotypic variation, while chloroplast DNA markers (matK and trnH–psbA) were used to assess genetic diversity and population structure. In parallel, sensory evaluation was conducted to examine how variation in fruit traits translates into human perception and preference. By linking genetic structure with morphological traits and consumer-relevant sensory attributes, this study moves beyond traditional single-dimension analyses. By integrating multiple dimensions of variation, this study contributes to a more holistic understanding of *M. zeyheri* and supports its transition from a wild-harvested resource to a strategically developed indigenous fruit crop.

## 2. Materials and Methods

### 2.1. Morphological Analysis

#### 2.1.1. Morphological Sample Collection and Analytical Procedure

Morphological data were obtained from *M. zeyheri* trees growing in the same sites where leaf samples for DNA analysis were collected, namely Vhembe District of Limpopo and Ehlanzeni District of Mpumalanga. In each region, twenty trees were selected to correspond with those sampled for molecular work. From every individual, representative samples of fruits, leaves, and bark were collected. Mature leaves and fully ripened fruits were chosen to ensure comparability between regions and to minimize developmental bias. Bark characteristics were not sampled destructively; instead, high-resolution photographs of the bark were taken in situ to allow for morphological comparison between study sites. After collection, the fruits were placed in labelled paper bags, transported to the laboratory, and processed within seventy-two hours to minimize shrinkage and moisture loss. In the laboratory, fruits were washed under running distilled water to remove dirt and debris, air-dried on absorbent paper, and then manually dissected with a sterile stainless-steel scalpel to separate the nuts from the pulp. Each fruit and its corresponding nut were assigned unique identification codes to prevent mix-ups. Morphological measurements were performed using a MAC AFRIC 150MM digital Vernier calliper (Adendorff, Johannesburg, South Africa) with an accuracy of ±0.01 mm. Fruit length was measured as the distance from the apex to the base of the fruit, while fruit width was taken as the maximum equatorial diameter after rotating the fruit to locate its widest point. Nuts were cleaned and air-dried for twenty-four hours before measuring their maximum longitudinal size, expressed in centimetres. Leaf width was measured as the maximum distance across the leaf lamina to its apex, and length was measured from the base of the lamina to the apex along the midrib. Leaf area was estimated using leaf length and width measurements, adjusted by a correction factor of 0.66, obtained from established reported leaf area estimation approaches for broad non-rectangular leaves in a study by [11], where leaf area is approximated as the proportion of the product of length and width to account for taper and shape. All measurements were replicated thrice by the same collector to reduce observer bias, and the mean of the three measurements was recorded as the final value.

#### 2.1.2. Data Analysis

All quantitative data were first entered and cleaned in Microsoft Excel before being analysed using R (Version 4.3.2) and IBM SPSS Statistics Version 29 (Armonk, NY, USA). Descriptive statistics, including means, standard deviations, and coefficients of variation, were calculated for each trait to provide a summary of morphological variability within and between regions. Differences among populations (Limpopo vs. Mpumalanga) were tested using one-way analysis of variance (ANOVA). When significant differences were detected, Tukey’s HSD test was used for pairwise comparisons.

### 2.2. Sensory Evaluation

#### 2.2.1. Fruit Collection and Sensory Procedure

Fruits used for sensory evaluation were collected directly from the same *Mimusops zeyheri* trees sampled for morphological and genetic analyses in Vhembe District (Limpopo) and Ehlanzeni District (Mpumalanga). Only fully ripe, undamaged fruits with uniform colour and firmness were selected to maintain consistency across sites. After harvesting, fruits were rinsed with distilled water, air-dried, and stored in insulated coolers to preserve freshness during transport. Sensory sessions were conducted separately in each region, and participants were intentionally presented with fruits sourced from their own locality to avoid unfamiliarity effects. Before serving, fruits were brought to room temperature and cut into uniform bite-sized portions using sterilized utensils. Each portion was placed in identical, coded, non-transparent containers to minimize visual bias, and all samples were served in a randomized sequence.

The sensory assessment was carried out separately in Limpopo and Mpumalanga, with fifty willing participants recruited in each region. Participants were adults (≥18 years), and basic demographic information, including age group and gender, was recorded to assess potential sources of bias in sensory preferences. All participants took part voluntarily after being informed about the purpose and nature of the study. To improve objectivity and minimize visual bias, fruit samples were presented in closed, coded containers, preventing participants from making selections based on appearance prior to evaluation. Sensory evaluations were conducted in small groups under calm, well-lit conditions to avoid external distractions. Respondents were instructed not to discuss their impressions with others while scoring to maintain independent evaluations. A blind tasting protocol was applied in which samples were anonymized using random codes. Between samples, participants rinsed their mouths with clean water to reduce carry-over effects. Each participant evaluated eight sensory attributes (appearance, aroma, taste, sweetness, texture, mouthfeel, aftertaste, and overall acceptability) using a standardized 9-point hedonic scale, where 1 = “Dislike” and 9 = “Very Liked”. Completed score sheets were checked for completeness before collection. To ensure data quality, incomplete or inconsistent responses were excluded from analysis. In addition, any missing or failed observations (e.g., due to participant non-response or sample issues) were documented and handled using appropriate statistical procedures to avoid bias in the results.

#### 2.2.2. Data Analysis

Data from the sensory evaluation were entered into a cleaned spreadsheet and analysed using descriptive and inferential statistical procedures. Mean, standard deviation, minimum, and maximum values were calculated for each sensory attribute at each site. To assess differences between the two regions, Welch’s *t*-test was applied because it accounts for unequal variances and sample sizes. To complement significance testing with a measure of practical magnitude, Cohen’s d was calculated for each attribute as the standardized mean difference between regions, interpreted against conventional benchmarks for small (~0.2), medium (~0.5), and large (~0.8) effects. To formally test whether the overall multivariate sensory profile differed between regions rather than relying solely on univariate comparisons a permutational multivariate analysis of variance (PERMANOVA) was performed on the eight-attribute sensory matrix using Euclidean distance, with region as the grouping factor and 999 permutations. A radar chart was used to visualize attribute profiles across sites, while Principal Component Analysis (PCA) was performed to identify the attributes contributing most to variation in sensory perception. All analyses were carried out using R (Version 4.3.2), and graphical outputs were generated using the ggplot2 version 4.0.0, FactoMineR version 2.1.2, and factoextra packages version 1.0.7.

### 2.3. Genetic Analysis

#### 2.3.1. Leaf Sample Collection

**Figure 1:**
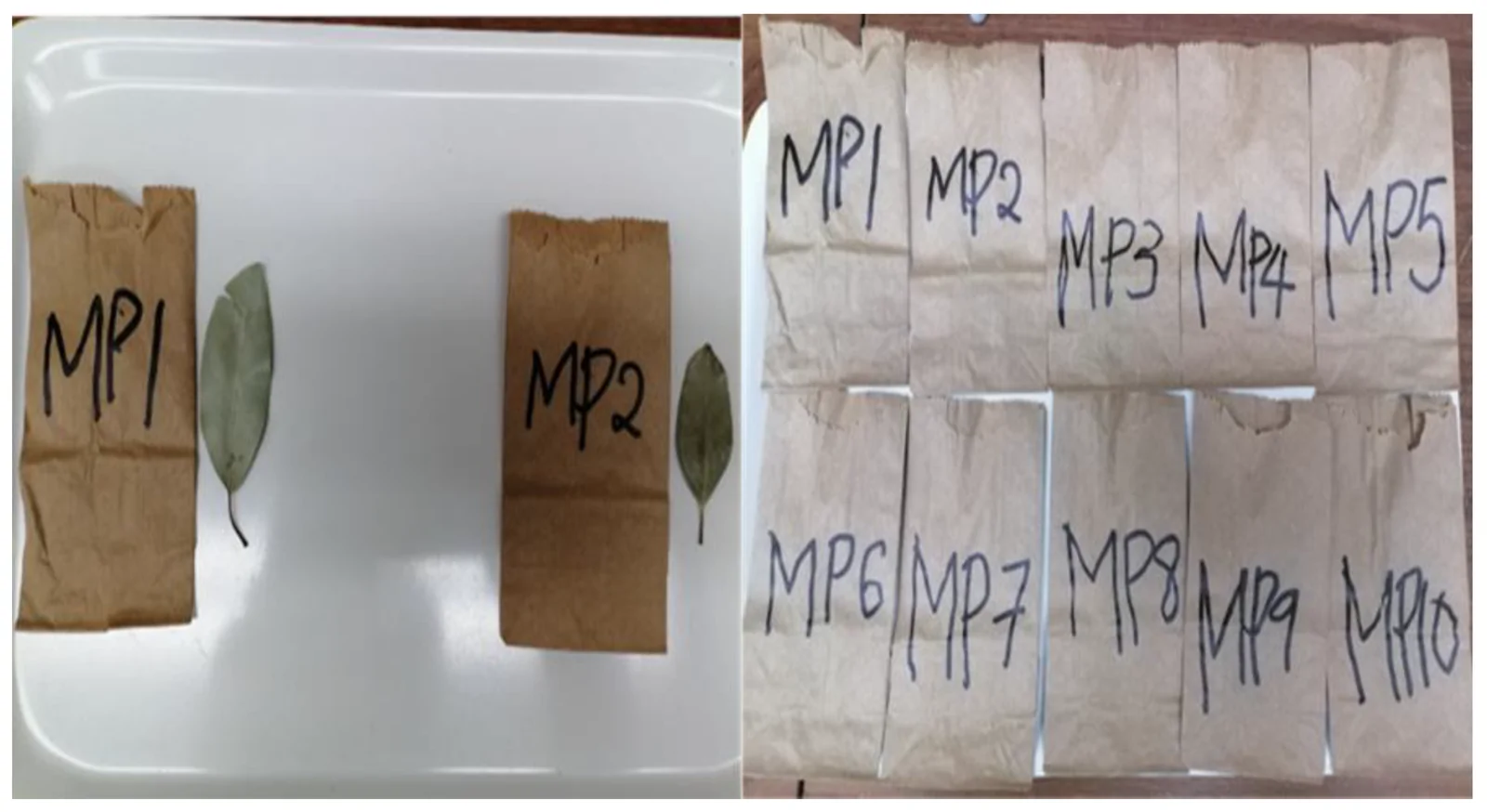
Collected leaf samples from Mpumalanga (photo by Mkhonto C., 2025). The labels Mp1–10 corresponds to Mpumalanga plant samples 1–10.

Leaf samples of *M. zeyheri* were collected from two distinct populations representing contrasting agro-ecological zones: Vhembe District in Limpopo Province and Ehlanzeni District in Mpumalanga Province. At each site, twenty individual trees were randomly selected, and one mature, healthy leaf was sampled from each tree, resulting in a total of forty samples. To avoid sampling from genetically identical ramets, trees were spaced at least 30 m apart. Each leaf sample was immediately placed in a separate brown paper bag labelled according to its collection site and number (M1–M20 for Mpumalanga and LP1–LP20 for Limpopo) as shown in Figure 1. The bags were kept in a portable cooler during transport from the field to the laboratory to minimize degradation of genetic material. Upon arrival, samples were air-dried and stored at 4 °C until DNA extraction and subsequent molecular analyses.

#### 2.3.2. DNA Extraction, Sequencing and Analysis

DNA analysis was performed at the University of Johannesburg African Centre for DNA Barcoding, Department of Botany and Plant Biotechnology. DNA extraction is an internationally recognized technique for extracting DNA from plant samples. It is a process of purification of DNA from a sample using a combination of chemical and physical methods. The DNA extraction was performed using the CTAB (cetyltrimethylammonium bromide) method The DNA extracted was processed using a Polymerase Chain Reaction (PCR), an internationally recognized technique used to amplify DNA fragments through a process of temperature cycling, thereby generating thousands to millions of copies of a particular DNA region. The core barcoding region matK and an additional gene trnH- psbA were selected for this test. Negative and positive controls were used to ensure the procedure’s correctness (i.e., no contamination was detected). All PCRs were performed using Amplicon (Ampliqon, Stenhuggervej, Denmark) TAQ DNA Polymerase Master Mix Red (www.amplicon.com, accessed 8 July 2025) with the addition of 3.2% bovine serum albumin. The instrument used for PCR was a Gene Amp^®^ PCR System 9800 Fast Thermal Cycler (Applied Biosystems, Foster City, CA, USA). PCR products were visualized on 1% agarose gels, and visible products were selected for DNA sequencing.

The amplified fragments were then bi-directionally sequenced using an internationally recognized method of Sanger cycle sequencing that provides the actual DNA code of the amplified regions. The method is based on the selective incorporation of chain-terminating dideoxy-nucleotides by DNA polymerase during DNA replication. Cycle sequencing was performed using the BigDye^®^ Terminator V3.1 (Thermo Fisher Scientific, Waltham, MA, USA) kit. The DNA code was visualized using a 3500 Genetic Analyzer (Thermo Fisher Scientific). Negative and positive controls were used to ensure the procedure’s correctness. Complementary strands were assembled, edited, and aligned using Geneious v.8.1.9 (Biomatters Ltd., Auckland, New Zealand). The sequence data for the targeted regions was analysed. The algorithm Basic Local Alignment Search Tool BLAST version 2.17.0 (https://blast.ncbi.nlm.nih.gov/Blast.cgi (accessed on 9 July 2025)) was used to compare query sequences to GenBank (NCBI https://www.ncbi.nlm.nih.gov/genbank (accessed on 9 July 2025)) and the Barcode of Life Database BOLD version 5 (https://www.boldsystems.org/ (accessed on 9 August 2025)), both of which are internationally recognized sequence databases. A BLAST search compares a query sequence with the library of sequences available in GenBank and identifies sequences that resemble the query sequence above a certain threshold.

## 3. Results

### 3.1. Morphology

#### 3.1.1. Bark Morphology

**Figure 2:**
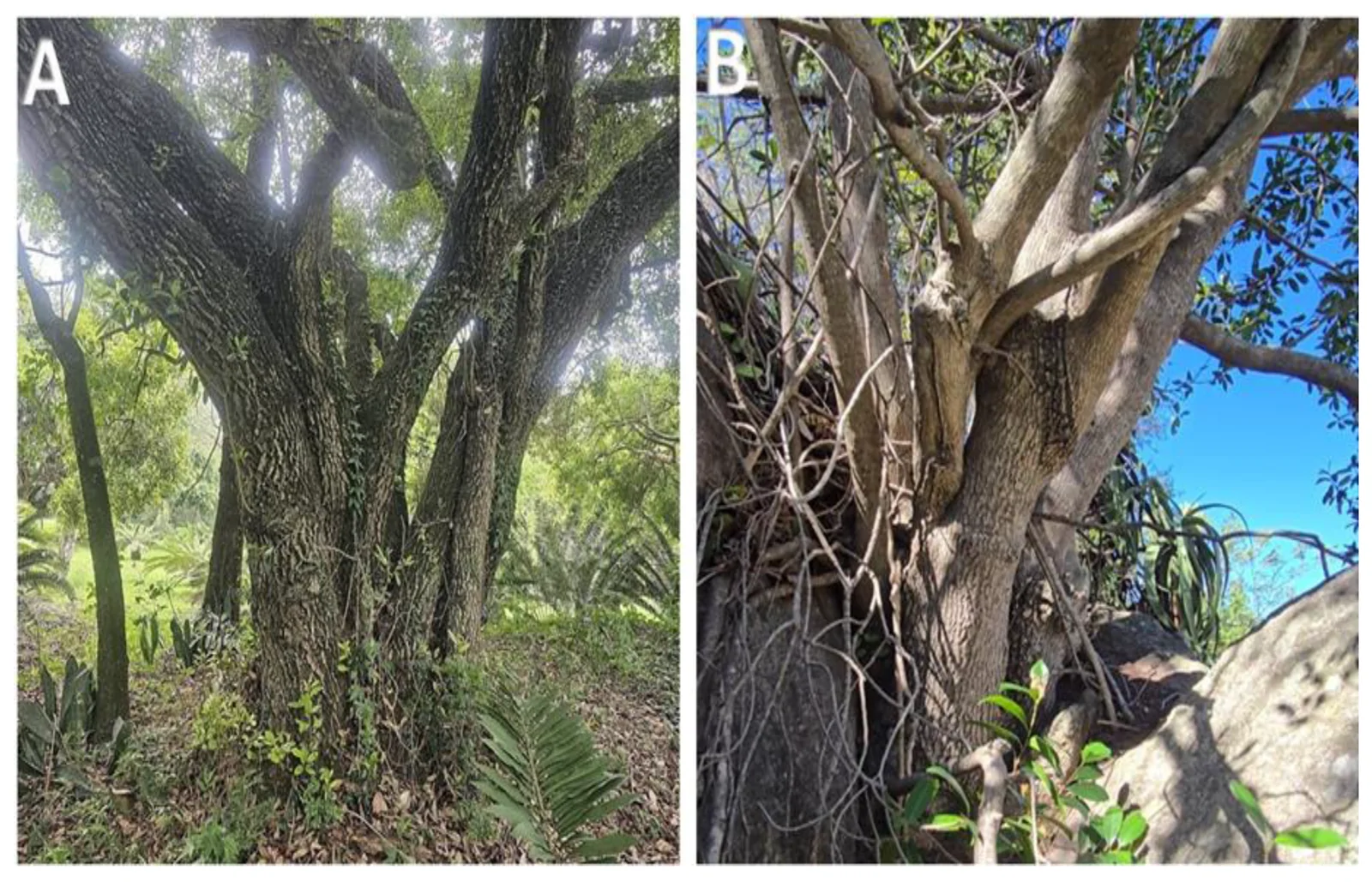
Comparative bark morphology of *M. zeyheri* populations from two regions in South Africa, Mpumalanga, Ehlanzeni District (**A**), and Limpopo, Vhembe District (**B**) (photo by Mkhonto C., 2025).

Bark characteristics were assessed qualitatively from field photographs rather than through direct quantitative measurement (bark thickness or fissure depth); the comparisons are therefore read as descriptive observations rather than measured traits. Bark morphology of *M. zeyheri* showed noticeable variation between the two study sites (Figure 2). In Mpumalanga, individuals were characterized by dark brown to blackish bark that was deeply fissured and coarse, with visible lichen growth on the bark surface. In contrast, trees from Limpopo exhibited lighter grey-brown bark that was comparatively thinner and smoother, with minimal fissuring.

#### 3.1.2. Nut and Fruit Morphology

**Figure 3:**
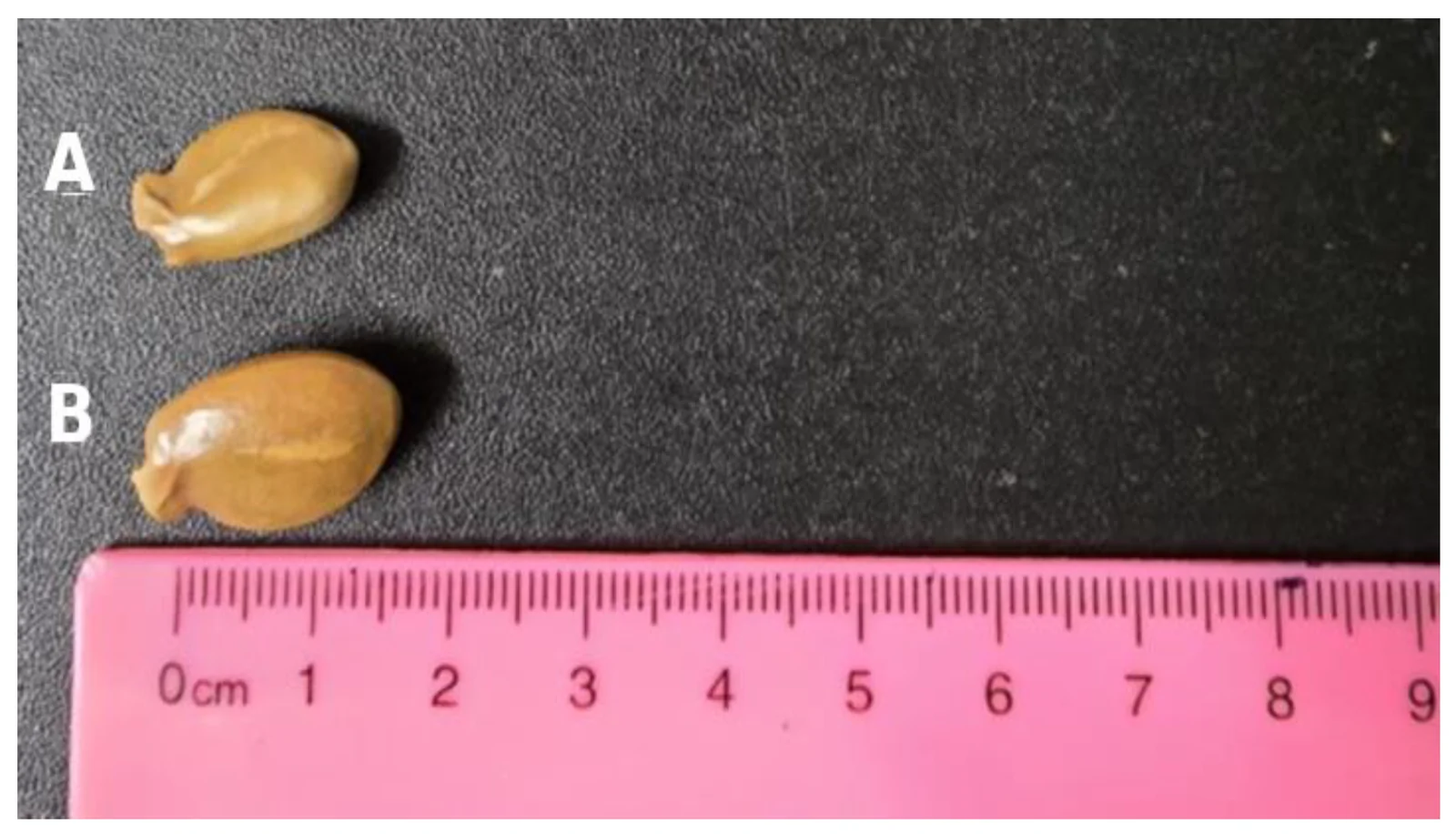
Comparison of *M. zeyheri* nut morphology from two regions in South Africa, Limpopo, Vhembe District (**A**) and Mpumalanga, Ehlanzeni District (**B**), (photo by Mkhonto C., 2025).

Nut and fruit size results of *M. zeyheri* from Limpopo and Mpumalanga are shown in Figure 3 and Table 1. The results revealed a clear and consistent pattern: across all reproductive traits measured, nut size, fruit length, and fruit width, the Mpumalanga population exhibits noticeably larger values than the Limpopo population. Mpumalanga individuals produced larger nuts (2.11 ± 0.36 cm) and larger fruits, both in length (29.41 ± 0.61 mm) and width (24.14 ± 0.55 mm), whereas Limpopo individuals had smaller nuts (1.81 ± 0.31 cm) and smaller fruit dimensions overall. Joint analysis of nut and fruit traits revealed a strong morphological distinction between the two regions, supporting the view that these populations differ in their overall reproductive phenotype. Although larger fruits and larger nuts align when the species is considered as a whole, the within-site correlations are weak (r = −0.328 between nut size and fruit length in Limpopo and r = 0.117 in Mpumalanga), indicating that individual trees do not consistently produce larger nuts when they produce larger fruits. Instead, the association between fruit and nut size appears to be structured at the population level.

#### 3.1.3. Leaf Morphology

**Figure 4:**
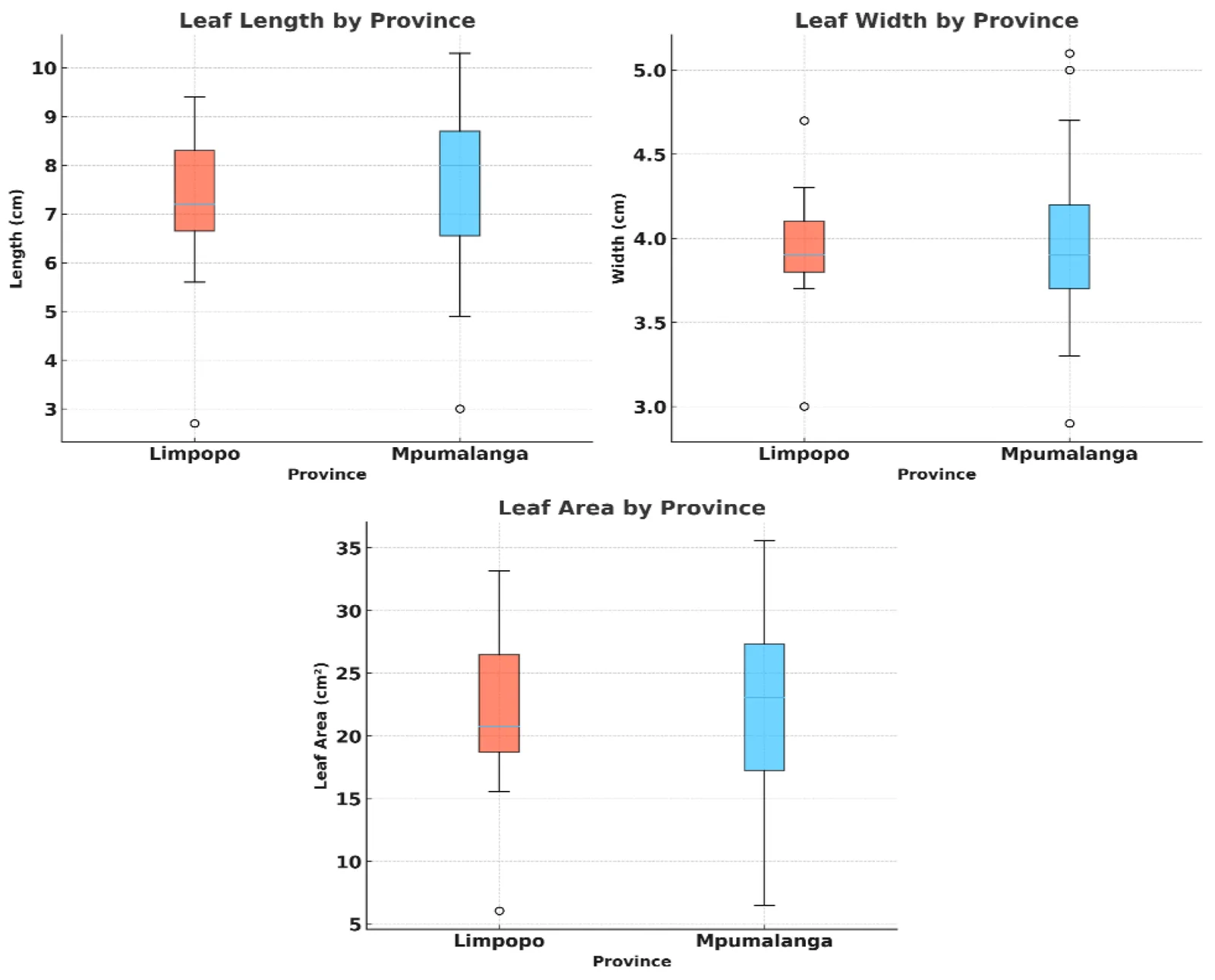
Variation in leaf length, leaf width, and estimated leaf area of *M. zeyheri* from Limpopo and Mpumalanga.

The leaf morphometric results in Figure 4 reveal minimal differences in leaf size between Limpopo and Mpumalanga populations of *M. zeyheri*. Mean leaf length was 7.23 cm in Limpopo and 7.52 cm in Mpumalanga, while mean leaf width measured 3.93 cm and 4.03 cm, respectively. Estimated leaf area also showed strong similarity, with averages of 21.69 cm^2^ (Limpopo) and 22.80 cm^2^ (Mpumalanga). The narrow range of differences, typically less than one centimetre, indicates substantial overlap in leaf morphology across the two regions.

**Figure 5:**
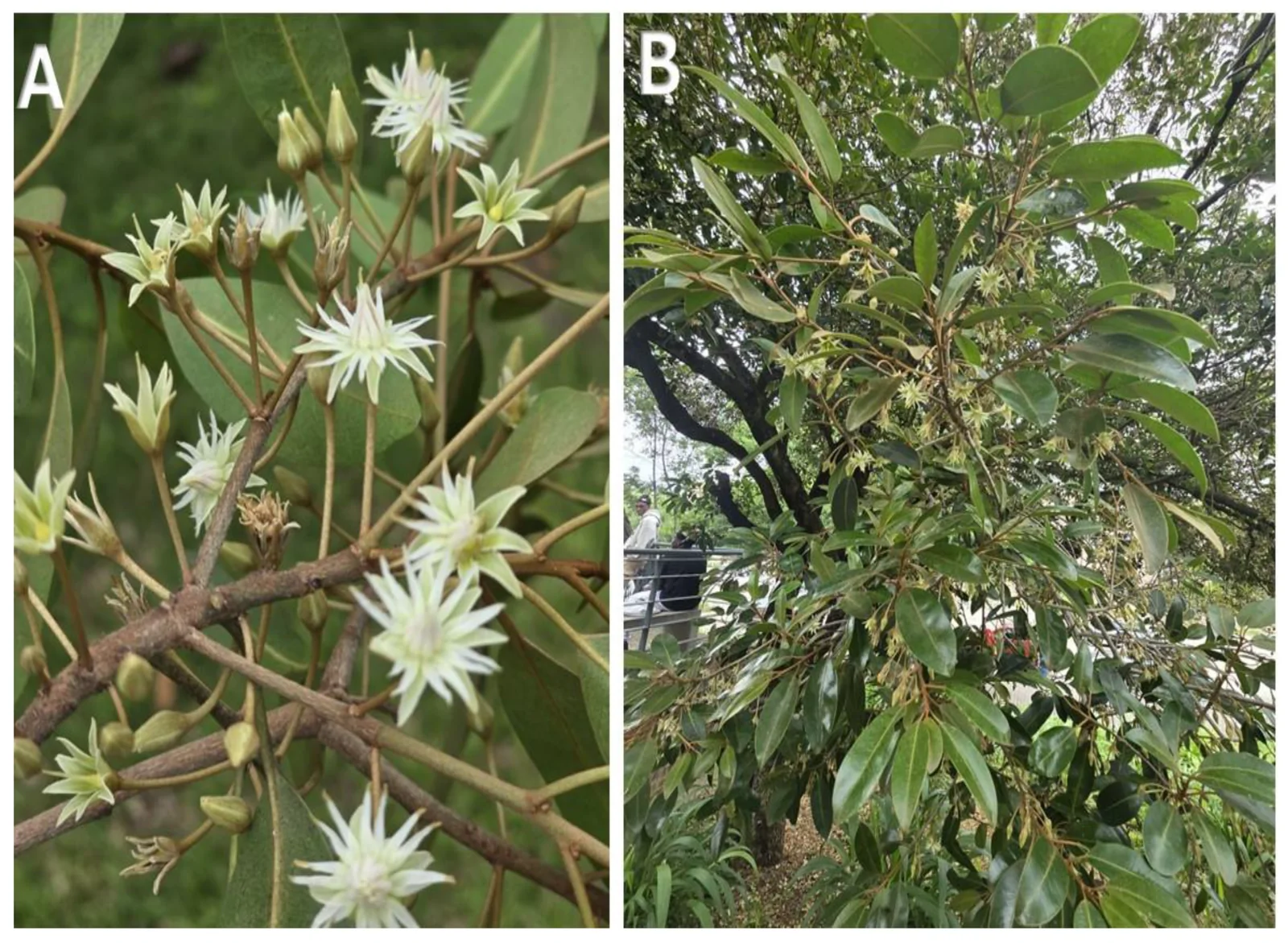
Early reproductive (**A**) and vegetative (**B**) morphology of *M. zeyheri* from the Mpumalanga populations (photo by Mkhonto C., 2025).

The Mpumalanga population of *M. zeyheri* displayed abundant reproductive activity during the sampling period, with numerous open flowers and developing buds observed along the axillary nodes (Figure 5A). Flowers exhibited the typical multi-segmented, star-shaped corolla characteristic of the species, with creamy-white tepals radiating symmetrically from the centre. Compared to Limpopo samples, Mpumalanga individuals carried significantly more flowering clusters per branch, suggesting favourable phenological conditions during the sampling interval. Vegetatively, the leaves were elliptic, glossy, and medium to dark green with entire margins (Figure 5B). No gall formation was observed in Mpumalanga trees, in contrast to several Limpopo individuals that exhibited widespread galling. Leaf size remained relatively constant across populations, with only slight variation, consistent with the quantitative measurements recorded in this study.

**Figure 6:**
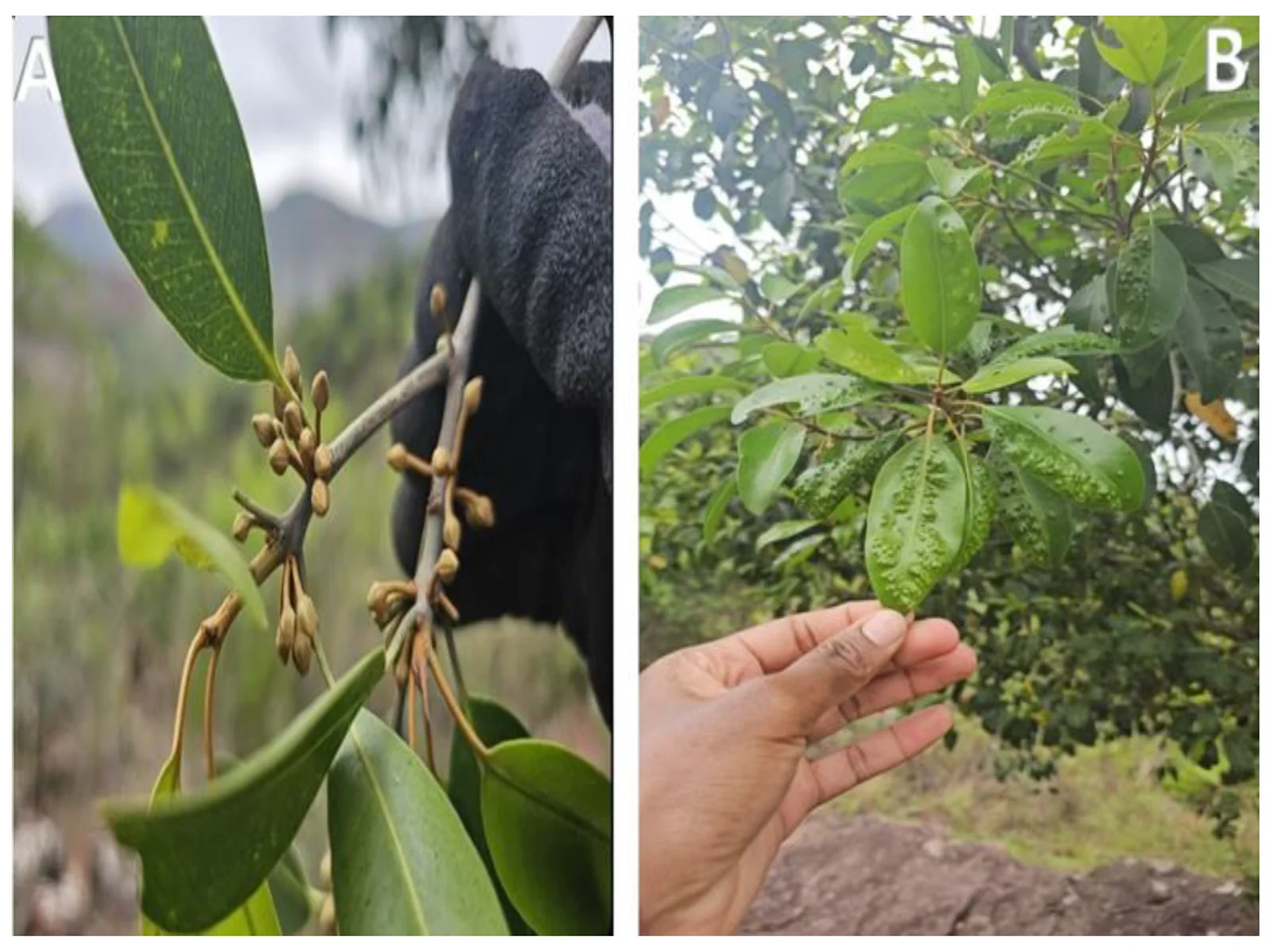
Early reproductive (**A**) and vegetative (**B**) morphology of *Mimusops zeyheri* from the Limpopo populations (photo by Mkhonto C., 2025).

The Limpopo population of *Mimusops zeyheri* showed visible signs of reproductive activity during the sampling period, with several branches bearing compact clusters of immature axillary flower buds (Figure 6A). This observation is based on field visual assessment rather than quantitative measurements of phenological stages such as days to flowering or fruiting. The presence of these structures indicates that individuals in this population were actively entering a reproductive phase at the time of sampling. This finding is presented as a descriptive observation and is not directly linked to the measured leaf morphological traits in this study.

These buds were small, oval, and tightly grouped, indicating that the trees were in the initial stages of floral initiation before anthesis. Vegetatively, leaves were elliptic, glossy, and medium green; however, a notable feature of the Limpopo samples was the presence of pronounced gall formation across the leaf lamina (Figure 6B). Although not quantified the observed insect-induced galling could plausibly affect leaf photosynthetic area and, indirectly, tree vigour and fruit development if severe; however, since gall density did not visibly alter leaf shape or venation in the sampled individuals, we treat this as a descriptive biotic-stress observation rather than a factor influencing the morphological or sensory results reported here. Quantifying gall incidence and its relationship to fruit yield or quality would be a useful direction for future monitoring.

### 3.2. Sensory Perecptions

#### Participants’ Sensory Responses to Fruit Attributes

The descriptive statistics in Table 2 and Table 3 show a distinct and uniform pattern in consumer evaluations of *M. zeyheri* fruits across the two study sites. Fruits sourced from Limpopo exhibited higher mean scores across all eight sensory attributes in comparison to those from MPE (Mpumalanga—Ehlanzeni). Various attributes, notably taste, aroma, mouthfeel, and overall acceptability, exhibit significant differences, with Limpopo, Vhembe (LV), fruits consistently scoring between 7.7 and 8.0, while MPE fruits range from 5.0 to 5.4. The 9-point scale (1 = very poor; 9 = excellent) highlights the extent of this difference; LV fruits are positioned between “very good” and “excellent,” whereas Mpumalanga, Ehlanzeni (MPE), fruits are nearer to “fair” or “average.” This indicates a significant difference in sensory appeal between the two geographical zones.

Beyond statistical significance, the magnitude of these regional differences was substantial. Welch’s *t*-tests confirmed significant differences between regions for every sensory attribute, with effect sizes (Cohen’s d) ranging from 2.14 to 3.34—two- to four-fold larger than the conventional benchmark for a “large” effect (d = 0.8) (Table 3). This indicates that the observed divergence in sensory perception between regions is unlikely to reflect sampling variability or measurement noise alone.

**Figure 7:**
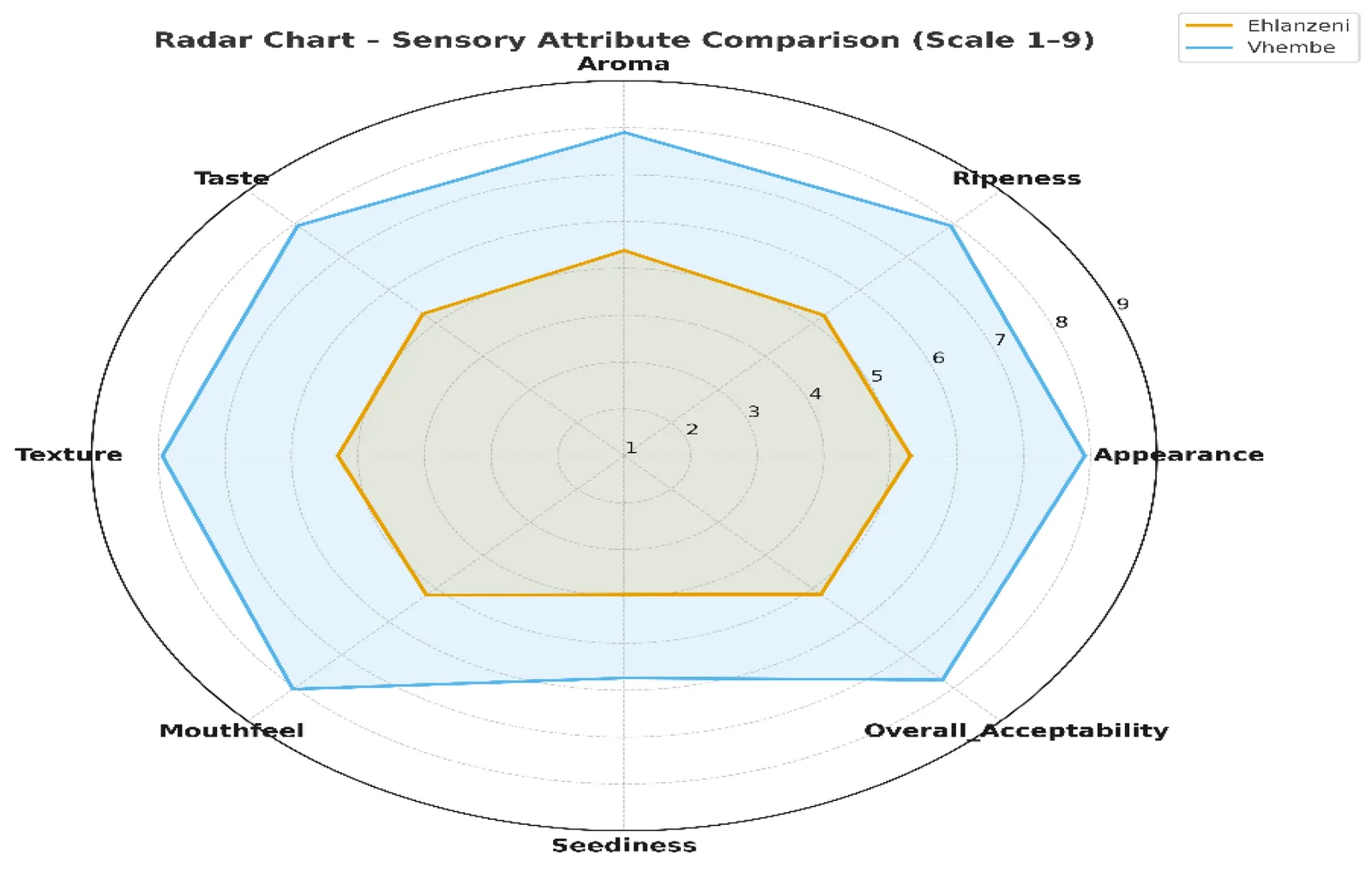
Radar plot showing the mean sensory evaluation scores of *Mimusops zeyheri* fruits across the two study sites, LV (Limpopo, Vhembe) and MPE (Mpumalanga, Ehlanzeni).

**Figure 8:**
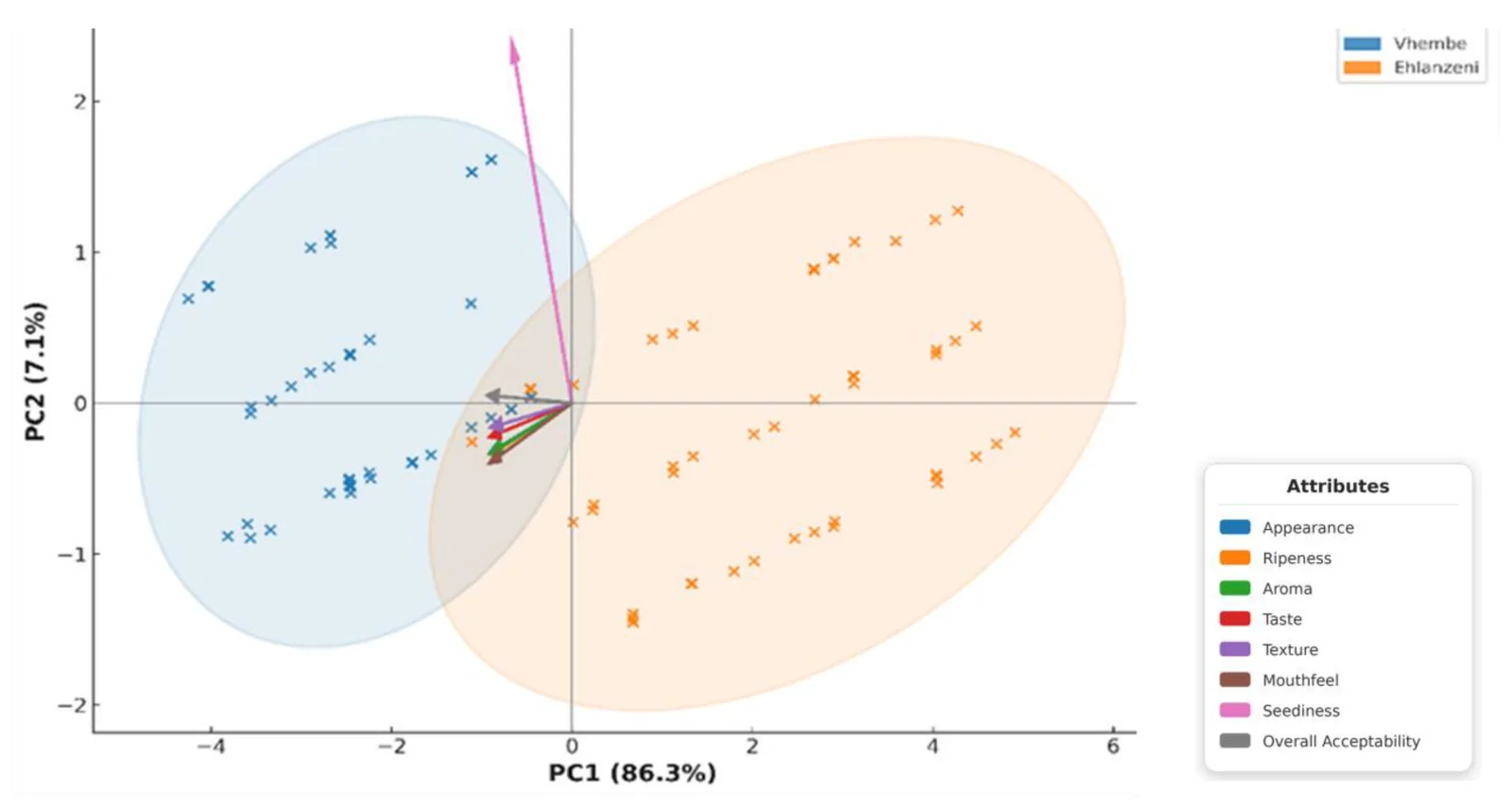
Principal Component Analysis (PCA) biplot illustrating respondents’ overall sensory perception patterns for *Mimusops zeyheri* fruits from LV (Limpopo, Vhembe) and MPE (Mpumalanga, Ehlanzeni). Points represent individual respondents; shaded ellipses depict 95% confidence regions for each study site. Colour-coded loading vectors represent the contribution of sensory attributes to PC1 and PC2, with corresponding attribute colours shown in the legend.

To formally test whether these univariate differences corresponded to a distinct overall sensory profile, a permutational multivariate analysis of variance (PERMANOVA) was performed on the eight-attribute sensory matrix using Euclidean distance, with region as the grouping factor (999 permutations). This confirmed a highly significant multivariate separation between regions (pseudo-F = 185.51, R^2^ = 0.654, *p* = 0.001), indicating that region accounts for approximately 65% of the total variance in the joint sensory profile across all eight attributes. This result corroborates the univariate and descriptive findings above, confirming that Limpopo and Mpumalanga fruits differ not only in individual attributes but in their overall sensory profile. The radar chart (Figure 7) and PCA (Figure 8) together provide a comprehensive understanding of how respondents from both sites perceived the sensory qualities of *M. zeyheri*. The radar chart offers a direct visual comparison of mean sensory scores across attributes, clearly illustrating differences in how the fruit was perceived between Limpopo and Mpumalanga. It highlights that samples from Limpopo consistently received higher ratings across most attributes, including appearance, aroma, taste, and mouthfeel, thereby providing an intuitive overview of sensory preference patterns. The PCA complements and extends these observations by illustrating how the sensory attributes collectively contribute to overall perception. Attributes that appeared more pronounced in the radar chart, particularly aroma, taste, and mouthfeel, also carried the strongest loadings in the PCA, confirming that these attributes were the primary drivers of the separation between the Limpopo and Mpumalanga samples in multivariate sensory space.

### 3.3. DNA Analysis

DNA barcoding was employed to assess the level of genetic diversity and relatedness among *Mimusops zeyheri* populations collected from Limpopo and Mpumalanga using two chloroplast markers, matK and trnH-psbA. These markers provide complementary information on sequence divergence and phylogenetic clustering, allowing the identification of genetic variation within and between populations. Voucher table showing PCR and sequencing results (Table 4). Of the 40 trees sampled for morphological and sensory analysis (20 per region), only 20 (10 per region, a 75% amplification/sequencing success rate from the original submissions) yielded usable chloroplast sequence data. As a result, the genetic dataset does not include the same full set of individuals evaluated for fruit, nut, and leaf morphology or sensory attributes, and the three datasets should not be assumed to represent identical trees. This incomplete overlap constrains the extent to which genetic lineage assignment can be directly attributed to specific morphological or sensory phenotypes at the individual level. It is worth noting that matK and trnH–psbA are maternally inherited chloroplast markers; they capture only maternal lineage history and cannot resolve biparental gene flow, nuclear genetic diversity, or adaptive variation. The ‘lineages’ and ‘clusters’ discussed below should therefore be interpreted as maternal-lineage groupings rather than as evidence of the species’ full genetic architecture. Nuclear markers such as microsatellites or SNPs would be required to assess the complete population structure.

#### 3.3.1. Sequence Alignment and Marker Efficiency

**Figure 9:**
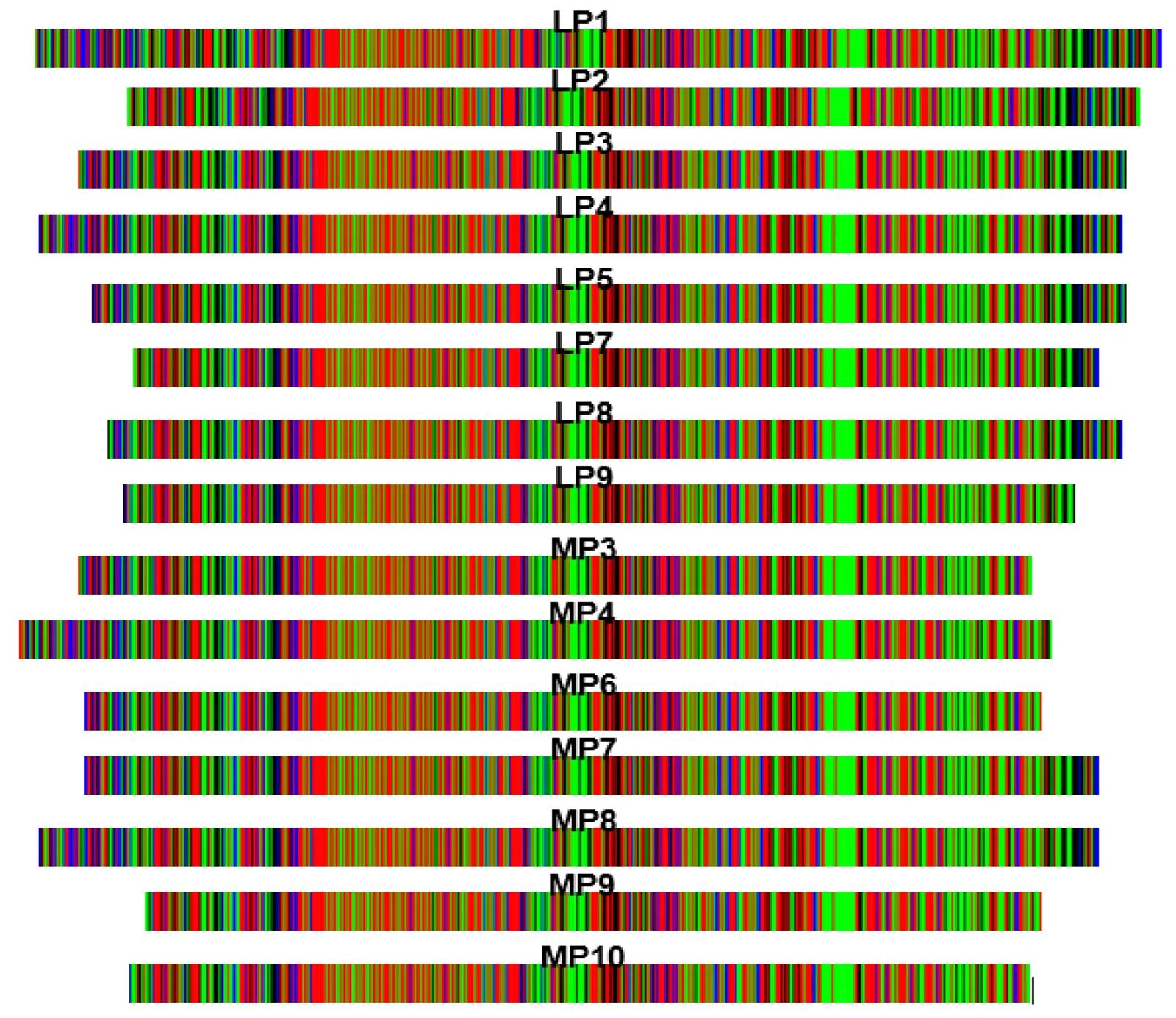
DNA barcode representation of *M. zeyheri* samples from Limpopo (LP) and Mpumalanga (MP) based on the trnH–psbA marker. Each horizontal bar represents an individual sample, with coloured segments indicating nucleotide variation along the sequence, thereby illustrating genetic similarities and differences among individuals. Colours follow the standard nucleotide convention used in Geneious and most Sanger sequencing trace viewers: green = adenine (A), red = thymine (T), blue = cytosine (C), and black = guanine (G). Samples sharing the same colour carry the same base, while a change in colour across samples at that position marks a nucleotide substitution; where bar segments differ in width, this reflects an insertion or deletion relative to the consensus sequence. Columns of uniform colour running across all samples represent conserved, invariant sites, whereas columns with mixed colours mark polymorphic sites that distinguish individuals or populations.

Figure 9 presents the DNA barcode profiles of *M. zeyheri* individuals from Limpopo (LP) and Mpumalanga (MP), generated using the chloroplast trnH-psbA intergenic spacer. Each horizontal bar corresponds to a single sample, with colours representing the different nucleotides (A, T, C and G) at aligned positions along the sequence. The distinct vertical colour shifts indicate polymorphic sites, while uniform colour stretches denote conserved regions. Samples LP1–LP9 and MP3–MP10 exhibit clear sequence variation, with several accessions showing unique colour blocks, indicating nucleotide substitutions, insertions, or deletions relative to the consensus sequence.

#### 3.3.2. Genetic Distance and Clustering

**Figure 10:**
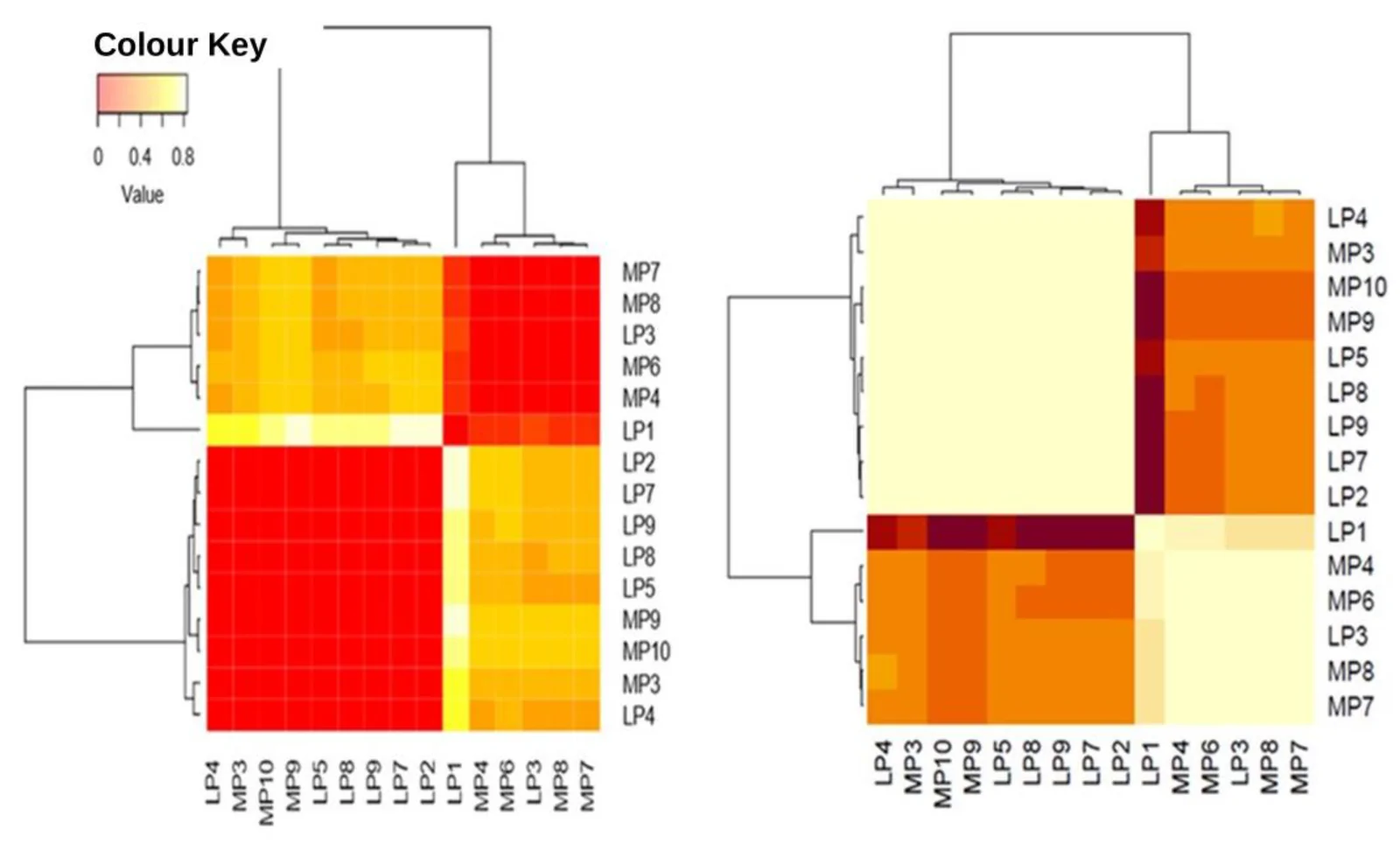
Heatmap showing pairwise genetic distances among *Mimusops zeyheri* samples from Limpopo and Mpumalanga based on combined chloroplast sequences. LP1–10 = all the Limpopo samples, while MP1–10 = all the Mpumalanga samples.

The heatmaps and clustering thresholds in Figure 10 provide a coherent picture of how *M. zeyheri* individuals group genetically across the two regions. The patterns in Figure 9 show the species contains two broad genetic lineages when assessed with the trnH-psbA marker, but a finer, four-cluster structure when using matK at a much stricter distance threshold. In both cases, individuals sort into well-defined blocks of similarity, which correspond closely with the warm and cool colour gradients in the heatmaps. The presence of two singletons in the matK results (LP3 and MP5) suggests that certain individuals carry rare haplotypes that lie outside the main genetic groups [12]. The structure of the matK clusters, detected at an optimized threshold of 0.0005, implies a lineage split that is both shallow and subtle. The first two clusters each contain a mix of Limpopo and Mpumalanga samples, indicating that shared ancestry or historical gene flow still links the two regions. In contrast, the trnH-psbA marker, operating at a much broader threshold (0.256), partitions the dataset into two major clusters that align neatly with the contrasting blocks in the heatmap.

## 4. Discussion

### 4.1. Morphological Variation and Environmental Adaptation

The observed differences in bark morphology suggest site-specific morphological variation associated with contrasting environmental conditions between the two regions. Trees in Mpumalanga are typically found in more humid and shaded environments such as forest margins and moist valleys. In contrast, Limpopo populations occur in relatively drier conditions, often associated with rocky outcrops and semi-arid savanna landscapes. These findings are consistent with patterns reported in the literature, where environmental factors influence bark characteristics such as fissuring, thickness, and pigmentation. For example, studies within Sapotaceae indicate that greater bark fissuring, thicker periderm layers, and darker pigmentation are often associated with higher moisture availability, enhanced cambial activity, and sustained secondary growth [13,14]. Conversely, lighter and smoother bark has been linked to drier environments, where reduced fissuring and thinner bark may help limit water loss and reflect solar radiation.

These xeromorphic traits align with observations in other Sapotaceae genera, where species in drier habitats often develop thinner, less fissured bark as adaptive responses to heat and moisture stress [15,16]. Moganedi and Sibara [17] also highlight the importance of environmental factors like soil type, rainfall, and altitude in understanding morphological differences, emphasizing the influence of ecological gradients on regional variation. In general, the bark morphology of *M. zeyheri* exhibits considerable phenotypic plasticity, driven by environmental diversity throughout its distribution, aligning with patterns observed in the Sapotaceae family.

The association between fruit and nut size appears to be structured at the population level. This type of population-level divergence in reproductive traits, with relatively loose tree-level correlations, has also been reported for other African fruit trees in the Sapotaceae and related families, including *Vitellaria paradoxa*, *Sclerocarya birrea* and *Chrysophyllum albidum* [18,19,20], where fruit and nut characteristics cluster by region or stand rather than tightly scaling within individual trees.

Beyond the descriptive statistics, the size differences observed in *M. zeyheri* are consistent with broader patterns in indigenous African fruit trees, where intraspecific variation in nut morphology is shaped by reproductive strategy, maternal resource allocation and the ecological roles nuts play in regeneration [21,22]. Larger nuts, such as those characterizing the Mpumalanga population, typically contain more stored reserves, which can support stronger early nutling growth and improve survival under competitive or shaded conditions. This trend has been documented in Vitellaria paradoxa and Sclerocarya birrea, where heavier nuts tend to give rise to seedlings with greater initial vigour and resilience [18]. Nut size differences in *M. zeyheri* may also influence germination patterns. Research on other Sapotaceae, including *Chrysophyllum albidum*, shows that large-nut morphotypes often germinate more slowly but sustain emergence over a longer period, whereas smaller nuts may germinate more rapidly, reducing the window of vulnerability to granivores and fungal pathogens [23]. This suggests that the larger nuts in Mpumalanga may favour robust but possibly slower-establishing seedlings, while the smaller nuts in Limpopo may support quicker germination and a faster response to transient recruitment opportunities.

At the same time, the smaller nuts typical of the Limpopo population should not be viewed as ecologically inferior. Numerous studies highlight the advantages of small nuts: they are generally produced in higher numbers, disperse more widely, and are more easily transported by birds and small mammals [24,25]. For fleshy-fruited trees that depend heavily on animal-mediated dispersal, such as *M. zeyheri*, smaller nuts may enhance colonization potential by reaching a broader range of microsites, including gaps and disturbed patches. The coexistence of these contrasting nut-size strategies within a species is well documented in savanna trees, where recruitment success is shaped not by a single “optimal” nut size but by trade-offs between dispersal, establishment and maternal investment [26]. In this context, the variation observed in *M. zeyheri* is consistent with the wider botanical literature, suggesting that both nut types contribute to population persistence through different but complementary roles within the regeneration niche. The documented fruit size differences reinforce this interpretation. Larger fruits in Mpumalanga provide greater pulp rewards, which are likely to be attractive to frugivores capable of longer-distance dispersal, while the associated larger nuts enhance early nutling performance [27]. In contrast, Limpopo’s smaller fruits and nuts may represent a strategy that stresses fecundity and dispersal breadth: smaller propagules, produced in larger numbers, can be handled by a wider array of dispersers and spread across more heterogeneous environments. These dynamics mirror classical nut-dispersal theory, which emphasizes the balance between nut size, dispersal potential and nutling performance as a central component of plant reproductive ecology [28,29,30].

The narrow range of differences, typically less than one centimetre, indicates substantial overlap in leaf morphology across the two regions. This pattern of morphological stability is consistent with findings from the broader leaf-trait ecology literature, where evergreen tropical species often exhibit conserved leaf dimensions across sites with comparable environmental conditions [31,32]. *Mimusops zeyheri*, a sclerophyllous species in Sapotaceae, is characterized by thick, leathery leaves that maintain structural consistency across environmental gradients [33,34]. The slight tendency toward larger leaves in Mpumalanga may relate to marginally higher local moisture availability, as leaf size is known to respond to microclimatic conditions such as rainfall and temperature [35,36]. However, the differences observed here are small and fall well within expected intraspecific variation for African woody species.

Importantly, the considerable overlap in leaf area distributions suggests that both populations occupy a shared morphospace, reinforcing the ecological interpretation that leaf size in *M. zeyheri* is a highly stable functional trait. This aligns with global analyses showing that key morphological traits often exhibit limited divergence across populations unless subjected to strong selective pressures or highly contrasting climates [37]. Thus, the results indicate that *M. zeyheri* maintains consistent leaf morphology across its range in Limpopo and Mpumalanga, with only subtle, non-adaptive variation.

Regarding the contrasting reproductive and biotic-stress observations between sites, the more abundant flowering recorded in Mpumalanga suggests favourable phenological conditions during the sampling interval, while the pronounced gall formation observed in several Limpopo individuals, likely insect-induced, represents a descriptive observation of moderate biotic stress rather than a quantified effect; it is not directly linked to the measured leaf morphological traits in this study and did not visibly distort leaf shape or venation.

### 4.2. Sensory Perception and Regional Preference

These differences in sensory perception and regional preference reflect more than just inherent fruit quality; they echo how communities interact with wild fruits in their daily lives. Vhembe is a region where wild edible fruits are still widely collected, traded informally, and incorporated into the diets of rural households. Greater cultural familiarity often translates into more positive sensory assessments, as individuals develop an appreciation for flavour nuances, textural expectations, and ripeness indicators over time. It is important to note, however, that participants in this study tasted fruit only from their own region: Limpopo participants evaluated only Limpopo fruit, and Mpumalanga participants evaluated only Mpumalanga fruit. Consequently, the consistently higher scores recorded for Limpopo fruit cannot be unambiguously attributed to intrinsic fruit quality—regional familiarity, cultural preference, and expectation bias are plausible alternative explanations that this design cannot rule out. A blind cross-over design, in which participants from both regions evaluate fruit from both regions, would be required to separate genuine quality differences from familiarity effects, and this is recommended for future work; the sensory findings reported here should accordingly be read as regional preference patterns rather than as demonstrated differences in intrinsic fruit quality.

Overall, respondents from both study sites rated fruit favourably across several attributes, indicating that the species possesses generally appealing sensory characteristics. Attributes such as appearance, aroma, taste, and overall acceptability received moderate-to-high scores in both Vhembe and Ehlanzeni, reflecting a broad appreciation of the fruit’s quality across regions. Although Vhembe consistently recorded slightly higher mean values, the overall shape of the profiles suggests that both respondent groups recognized *M. zeyheri* as a palatable fruit. Across both sites, aroma emerged as one of the key sensory drivers of acceptance. The literature consistently shows that aroma plays a decisive role in shaping consumer expectations because it results from the combined effect of numerous interacting volatile organic compounds [38]. In this study, aroma scores differed only slightly between the two sites, yet the attribute remained influential for both groups. Similar findings have been reported in sensory studies on underutilized fruits such as *Hippophae salicifolia*, where aroma variations contributed to subtle differences in overall liking across products processed into juice, jam, or chutney [39]. These patterns emphasize that even modest differences in aroma intensity can shape how consumers perceive freshness and flavour richness.

Appearance played an important role in shaping how respondents from both sites viewed the fruit. Colour and surface characteristics are widely recognized as early indicators of ripeness and quality compounds [38,39] and this was reflected in the similar and generally positive scores for appearance in both Vhembe and Ehlanzeni. Comparable findings have been reported in other wild fruit studies, such as *Elaeagnus umbellata*, where appearance remained a stable attribute across different localities [40,41]. Texture and mouthfeel also contributed meaningfully to overall acceptability. Attributes such as firmness, juiciness and fibrousness are known to influence preference in both fresh and processed fruits [42]. In this study, both sites rated these attributes relatively well, with Vhembe showing slightly higher values. This indicates that the samples retained the tactile qualities typically associated with ripe *M. zeyheri*.

It is worth noting that the way the fruit was prepared for evaluation may have influenced how aroma and texture were perceived. Handling steps, such as cutting or short exposure to air, can influence the release of volatile compounds and surface moisture. Similar effects have been documented in sensory work on *Hippophae salicifolia*, where different processed forms showed varied sensory outcomes [39]. These preparation-related factors may explain some of the smaller differences observed between the two sites, while both still recognize *M. zeyheri* as a fruit with appealing sensory traits. Furthermore, maturity and harvest timing are crucial factors that greatly affect the perceived flavour, aroma, and texture. A study by Ilhan [42] highlights that soluble solid content and flavour compounds undergo substantial changes as the fruit matures, which influence the senses in individuals. The observed patterns suggest that sensory scores result from a complex interaction among biological, environmental, and experiential factors, rather than being exclusively dictated by chemical composition.

Beyond cultural familiarity, the consistently higher sensory scores of Limpopo fruits may also have a biochemical basis. Fruit flavour in many Sapotaceae is strongly influenced by the sugar-to-acid ratio and by the profile of volatile aroma compounds such as esters, terpenes, and aldehydes, both of which are sensitive to environmental stress during fruit development. Limpopo’s drier, more thermally variable savanna conditions could plausibly induce greater accumulation of soluble sugars and secondary metabolites relative to the moister Mpumalanga sites, a pattern documented in other water-stressed fruit crops where mild drought stress concentrates sugars and enhances aromatic compound synthesis. We did not measure sugar content, titratable acidity, or volatile compounds directly, so this explanation remains a hypothesis; instrumental analyses (e.g., HPLC for sugars/acids, GC-MS for volatiles) would be needed to test it directly. A key limitation of the sensory evaluation is that participants tasted fruit only from their own region: Limpopo participants evaluated only Limpopo fruit, and Mpumalanga participants evaluated only Mpumalanga fruit.

### 4.3. Genetic Structuring and Its Ecological Implications

The concentration of variable sites in LP3, MP5, and MP8, as indicated by more frequent colour changes, suggests the presence of private alleles or micro-haplotypes associated with specific locations. Similar patterns are reported in studies of Chrysophyllum albidum and Manilkara zapota, where geographically isolated populations carried distinct chloroplast variants linked to differences in climate and soil [43,44]. The environmental contrast between Limpopo’s dry savanna and Mpumalanga’s wetter Highveld could be driving similar selective pressures that shape chloroplast diversity in *M. zeyheri*, especially since chloroplast genes often play roles in stress tolerance, photosynthetic performance, and metabolic control [45]. It is worth noting that the level of barcode variation fits with known difficulties in getting clean DNA from Sapotaceae species [46]. Their tissues contain latex, polysaccharides, and phenolic compounds that interfere with DNA extraction, hampering PCR and increasing the chances of sequence errors or incomplete reads [46,47]. Even with these challenges, the consistent polymorphic patterns across samples suggest that the sequences represent genuine genetic differences rather than technical issues. Successfully capturing meaningful variation demonstrates that trnH-psbA holds up well even in tree species loaded with problematic secondary compounds [47].

The barcode also has ecological and biochemical relevance. Chloroplast variation can affect pathways involved in photosynthesis, carbon fixation, and the production of primary metabolites, which indirectly influence fruit nutrition [48]. Studies on *Vitellaria paradoxa* and *Pouteria campechiana* revealed that populations with different chloroplast haplotypes also exhibited differences in fruit phenolics, carotenoids, and antioxidant levels [49,50]. In *M. zeyheri,* it is possible, though entirely untested here, that the connection between barcode differences and regional sensory variations reflects chloroplast genetic structure contributing to the variation in fruit sweetness, aroma, and pulp colour documented in the morphological and sensory assessments; however, the sensory and genetic analyses were not performed on individually tracked, paired samples, so no direct link between genetic cluster and sensory outcome can be claimed from the present data.

The singleton cluster containing LP3 stands out, as such rare, outlying haplotypes have been documented in other Sapotaceae species, such as *Chrysophyllum albidum*, where isolated individuals harbour private mutations not yet diluted by cross-regional pollen flow [51]. This shift from four to two clusters when switching markers highlights how different genomic regions capture distinct layers of evolutionary history, one reflecting fine-grained, recent divergence, while the other captures deeper population structure [52,53].

The internal cohesion of these clusters raises important questions about the processes sustaining such patterns. In several Sapotaceae species, such as Pouteria torta and *Synsepalum dulcificum*, restricted nut dispersal has resulted in similarly compact genetic blocks, where offspring rarely move far beyond the maternal tree [54,55,56]. If *M. zeyheri* relies on frugivores with short foraging ranges, this could explain why lineages remain tightly knitted within sites. Over time, this restricted movement affects more than just genetics: it limits the spread of advantageous traits, constrains mating opportunities, and encourages the persistence of localized trait complexes linked to growth rhythm, fruit chemistry, and nutling vigour [56].

Such structuring also has implications for the tree’s phytochemistry and overall performance. Studies on species like Chrysophyllum viridifolium have shown that populations with narrow genetic bases tend to exhibit more uniform secondary metabolite profiles, often influencing their resistance to pests or environmental stress [57]. It is possible, though untested, that each maternal lineage of *M. zeyheri* carries its own phytochemical “signature” that could translate into regional differences in fruit qualities such as sweetness, aroma, or antioxidant capacity patterns that were also hinted at, though not confirmed, in the sensory evaluations. This is consistent with the report by Moore and Andrew [58], who further emphasize that high intraspecific variation provides plants with the capacity to evolve rapidly, suggesting that genetic diversity is crucial for adaptive responses to environmental challenges. It is worth noting that no direct association has been established in this study between specific chloroplast haplotypes and sensory scores, as the genetic and sensory analyses were not performed on the same individuals, and chloroplast markers are not themselves involved in flavour biosynthesis. The link proposed above is therefore a hypothesis for future testing, not a demonstrated result. Future studies using genome-wide association studies (GWASs) or quantitative trait locus (QTL) mapping on nuclear markers, applied to individually tracked trees with paired sensory and biochemical data, would be needed to establish whether specific genomic regions are truly associated with flavour traits.

The heatmap, therefore, provides more than an abstract genetic picture pointing to underlying biochemical and ecological differentiation with real consequences for how these trees cope with their environments. Finally, the separation observed between clusters suggests that conservation and utilization strategies should treat the two groups as distinct genetic resources. Lessons from *Manilkara multifida* and *Pouteria reticulata* show that mixing genetically divergent lineages can, under some circumstances, reduce nutling survival or disrupt locally adapted traits [59,60]. Preserving both lineages of *M. zeyheri* is therefore crucial, not only to safeguard genetic diversity but also to maintain the full spectrum of nutritional and physiological traits the species offers. The heatmaps, when viewed in this light, underscore the importance of region-specific management and highlight promising avenues for linking genetic structure with phytochemical potential, fruit quality variation, and long-term population stability.

## 5. Conclusions

This study set out to determine how *M. zeyheri* varies genetically and phenotypically across regions and how this variation relates to taste and consumer preferences. The results show that regional differentiation in *M. zeyheri* is driven jointly by environmental gradients (reflected in fruit/nut size and bark morphology) and by historical genetic divergence (reflected in distinct chloroplast lineages), and critically that sensory quality is decoupled from fruit size: the larger-fruited Mpumalanga population was consistently rated lower for taste and overall acceptability than the smaller-fruited Limpopo populations. Several gaps temper the interpretation of these findings. Morphology was limited to external traits, leaving the chemical basis of sensory differences unexplored. Sensory scoring, though useful, was not supported by instrumental assessments of sugars, volatile compounds, or secondary metabolites. The molecular analysis relied solely on chloroplast markers, which capture only maternal inheritance and do not fully resolve population structure or adaptive variation. Additionally, the study was confined to two provinces, yet *M. zeyheri* occurs across a wider environmental zone that may hold additional diversity. The sample size of 40 trees, while adequate for exploratory analysis, may limit the extent to which the findings can be considered a full representation of the species across its range.

Future work should extend genetic analyses to nuclear markers or genome-wide SNPs, incorporate detailed phytochemical profiling, and broaden geographic sampling, including increased sample sizes, to establish a fuller understanding of variation within the species. For conservation and utilization planning, the two primary maternal lineages identified, together with the rare haplotypes detected within them, should be recognized as genetically distinct units and managed accordingly in future conservation and breeding programmes. Domestication efforts may benefit from combining the favourable sensory traits prevalent in Limpopo with the larger fruit size observed in Mpumalanga, supporting the development of high-quality, climate-resilient cultivars.

Overall, the findings highlight the importance of adopting an integrative approach when evaluating underutilized fruit species. The clear decoupling between fruit size and sensory quality underscores the need for multi-trait selection strategies in domestication programmes. By combining the more favourably rated sensory attributes observed in Limpopo populations with the larger fruit size characteristic of Mpumalanga populations, there is strong potential to develop improved cultivars with both high consumer appeal and agronomic value. These insights position *M. zeyheri* as a promising candidate for sustainable crop development, contributing to food security, rural livelihoods, and climate-resilient agricultural systems.

## Figures and Tables

**Table 1:** Nut size and fruit morphological traits of *Mimusops zeyheri* from Mpumalanga and Limpopo, including correlations between nut size and fruit dimensions.

Province	Nut Length (mm)	Fruit Length (mm)	r (Nut–Fruit Length)	Fruit Width (mm)	r (Nut–Fruit Width)
Mpumalanga	2111 ± 0.36 ᵃ	29.41 ± 0.61 ᵃ	0.117	24.14 ± 0.55 ᵃ	−0.078
Limpopo	18.1 ± 0.31 ᵇ	27.41 ± 0.47 ᵇ	−0.328	23.22 ± 0.36 ᵇ	−0.063

Note: Values represent mean ± standard deviation. Different superscript letters indicate statistically significant differences between provinces (*p* < 0.05) according to Tukey’s multiple range test. r represents the Pearson correlation coefficient, indicating the strength and direction of the linear relationship between nut size and fruit dimensions.

**Table 2:** Summary statistics for *M. zeyheri* fruit sensory evaluation from Mpumalanga and Limpopo.

Attribute	LV Mean	MPE Mean	Diff	LV Std	MPE Std	LV Min	MPE Min	LV Max	MPE Max
Appearance	7.92	5.30	2.62	0.78	1.23	6	3	9	8
Ripeness	7.94	5.24	2.70	0.84	1.10	6	3	9	7
Aroma	7.90	5.38	2.52	0.71	1.18	6	3	9	8
Taste	7.94	5.28	2.66	0.71	1.14	7	3	9	7
Texture	7.94	5.30	2.64	0.74	1.15	7	4	9	8
Mouthfeel	8.04	5.20	2.84	0.78	1.16	7	3	9	8
Nuttiness	5.74	3.96	1.78	0.83	0.83	5	3	7	5
Overall Acceptability	7.76	5.18	2.58	0.62	0.90	7	4	9	7

Note: LV = Limpopo; MPE = Mpumalanga; Diff = difference between mean scores of Limpopo and Mpumalanga; std = standard deviation; Min = minimum observed score; Max = maximum observed score. Table 2 reports the mean, standard deviation, minimum, and maximum values for each attribute, together with the mean differences between sites. Sensory ratings were captured using a 9-point hedonic scale, where 1 indicated “very poor” and 9 indicated “excellent.” Comparisons between LV and MPE were performed using Welch’s independent-samples *t*-test, and the results indicated statistically significant differences (*p* < 0.05) for all attributes.

**Table 3:** Welch’s independent-samples *t*-test results and standardized effect sizes (Cohen’s d) for sensory attribute comparisons between Limpopo (LV) and Mpumalanga (MPE).

Attribute	t	df	*p*	Cohen’s d
Appearance	12.71	82.7	3.9 × 10^−21^	2.54
Ripeness	13.79	91.9	4.3 × 10^−24^	2.76
Aroma	12.98	80.3	2.0 × 10^−21^	2.60
Taste	13.96	82.0	2.2 × 10^−23^	2.79
Texture	13.67	83.7	4.8 × 10^−23^	2.73
Mouthfeel	14.35	85.9	1.6 × 10^−24^	2.87
Nuttiness	10.72	98.0	3.3 × 10^−18^	2.14
Overall Acceptability	16.70	87.5	6.1 × 10^−29^	3.34

Note: t = Welch’s t-statistic; df = Welch–Satterthwaite degrees of freedom; *p* = two-tailed significance level. Cohen’s d was calculated as the mean difference between regions divided by the pooled standard deviation.

**Table 4:** PCR amplification and sequencing success of *Mimusops zeyheri* samples from Limpopo (LP) and Mpumalanga (MP) using matK and trnH–psbA markers (green = successful amplification/sequencing; red = failed amplification/sequencing).

		PCR		Cycle Sequencing	
No	ID	maTK	trnH-psbA	matK	trnH-psbA
1	LP1				
2	LP2				
3	LP3				
4	LP4				
5	LP5				
6	LP7				
7	LP8				
8	LP9				
9	LP10				
10	LP11				
11	MP1				
12	MP2				
13	MP3				
14	MP4				
15	MP5				
16	MP6				
17	MP7				
18	MP8				
19	MP9				
20	MP10				

Note: Rows shaded green indicate successful amplification/sequencing; rows shaded red indicate failed amplification/sequencing. Only the 20 samples (10 per region) that were carried through to completion are shown; the remaining 20 samples collected did not yield usable PCR/sequencing results after two repeat attempts. Successful: 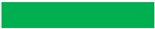; Unsuccessful: 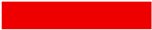.

## Data Availability

All the used data are contained in this article.

## Abbreviations

The following abbreviations are used in this manuscript:

ANOVA	Analysis of Variance
DNA	Deoxyribonucleic acid
LV	Limpopo (Vhembe)
MPE	Mpumalanga (Ehlanzeni)
PCA	Principal Component Analysis
PCR	Polymerase Chain Reaction
SD	Standard deviation
matK	maturase K gene
trnH–psbA	Chloroplast intergenic spacer
